# Draft genome sequence of *Burkholderia semiarida* isolated from *Theobroma cacao* in Chiapas, Mexico

**DOI:** 10.1128/mra.01377-25

**Published:** 2026-02-12

**Authors:** Nadia-Paola Ortiz-Conde, Leslie-Mariana Morales-Ruíz, Jeniffer-Chris Kerber-Diaz, Violeta Larios-Serrato, Paulina Estrada-de los Santos

**Affiliations:** 1 Departamento de Microbiología, Instituto Politécnico Nacional, Escuela Nacional de Ciencias Biológicas 603017 , Ciudad de México, México; 2 Departamento de Bioquímica, Instituto Politécnico Nacional, Escuela Nacional de Ciencias Biológicas 61735 , Ciudad de México, México; University of Manitoba, Winnipeg, Canada

**Keywords:** *Burkholderia*, *Theobroma cacao*, rhizosphere

## Abstract

During an investigation of *Burkholderia* species from agricultural soils in Mexico, *Burkholderia semiarida* was isolated from the rhizosphere of *Theobroma cacao* L. growing in Chiapas, Mexico. Here, we present the draft genome of *Burkholderia semiarida* CLM7-1.

## ANNOUNCEMENT

**Fig 1:**
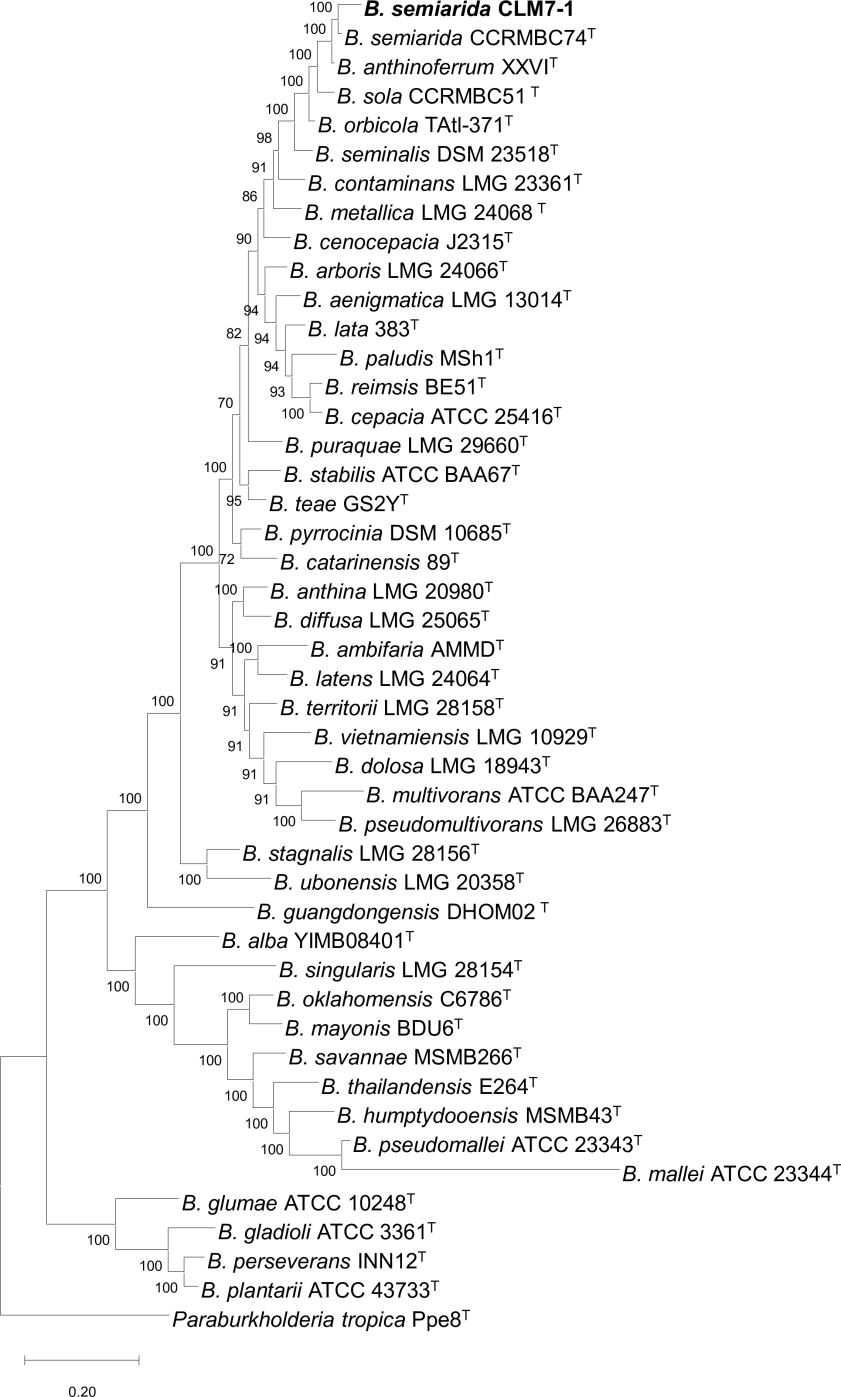
Phylogenomic tree of type strains of *Burkholderia* species and *Burkholderia semiarida* strains. The study used 400 conserved universal protein markers selected by PhyloPhlAn v 3.0 (13) for deep-branching phylogenies inferred by maximum likelihood. The amino acid substitution model (LG) and bootstrap analysis (1,000 replications) were performed using IQ-TREE (14). The bar corresponds to the number of differences between the sequences. In bold is indicated *B. semiarida* CLM7-1. The tree was generated using MEGA 10.

Through a study of *Burkholderia pseudomallei* in Mexico, several soil samples were collected along agricultural fields. The strain CLM7-1 was isolated from the cacao rhizosphere cultivated in Chiapas, Mexico (14°58′59.0″ N 92°09′10.0″ W). A sample of cacao rhizosphere (5 g) was resuspended in 5 mL sterile water, then 50 μL was streaked in Ashdown medium (1) and incubated at 37°C for 72 h. Since *B. pseudomallei* can present several colony morphologies (2), different colonies were selected. Among them, the CLM7-1 isolate was chosen and stored in 35% glycerol at –70°C. The isolate was grown in 5 mL BSE medium to obtain DNA for molecular identification and genome sequencing, which was purified using the CTAB protocol (3). The identification was carried out with universal primers 27F (5′-GTGCTGCAGAGACTTTGATCCTGGCTCAG-3′) and 1492R (5′-CACGGATCCTADGGGTACCTTACGACT-3′) (4) by amplifying the 16S rRNA gene with a Taq DNA Polymerase (New England Biolabs). The gene was sequenced using the SANGER technology at Macrogen INC. (https://www.macrogen.com/). The 16S rRNA gene sequence (PQ463740) identified the strain as *Burkholderia,* belonging to the *Burkholderia cepacia* complex (Bcc). The genome was sequenced by Novogene (https://www.novogene.com/us-en/) using the Illumina NovaSeq 6000 platform (150-bp paired-end reads; mean read length) with the NEBNext Ultra DNA library prep kit and an insert size of 350 bp. For all the analyses, default parameters were used except where otherwise noted. Raw paired-end reads were trimmed with Trimmomatic v0.39 (5) to remove low-quality sequences (<15 phred) and Illumina adapters. Quality assessment was performed through FastQC v0.12.1 (6). *De novo* assembly was conducted using MaSuRCA v4.0.8 (7). Annotation was done using the standard operating procedure at the NCBI Prokaryotic Genome Annotation Pipeline (PGAP) v5.1. The total genome length is 7,351,596 bp, containing 278 contigs, with an N50 of 134,978 bp, a genome coverage of 90×, completeness of 99.77%, and contamination of 0.94%. The G + C content is 67.18%, the total number of genes is 6,838, protein-coding genes 6,680, RNA genes 52, tRNAs 45, complete RNAs 1 5S, 1 16S, and 1 23S. Comparative genomics showed 98.5% similarity by average nucleotide identity (OrthoANIu) (8) and 87.4% similarity by digital DNA hybridization (Genome-to-Genome Distance Calculator 3.0) (9) with *B. semiarida* CBAS 905^T^. Phylogenomic analysis shows strain CLM7-1 clustering with *B. semiarida* CBAS 905^T^ (Fig. 1). This is the first isolation of *B. semiarida* in Mexico, a species described as causing onion-sour skin in Brazil (10) and recurrent pulmonary infection in an immunocompetent patient in China (11). However, this strain does not appear to cause damage to cacao, as the plants looked healthy at the time of soil sampling. Genome analysis in the antiSMASH 7.0 website (12) showed genes coding for the siderophore ornibactin (100%), the phenylpyrrole fungicide pyrrolnitrin (100%), and the metallophore aminopolycarboxylic acid (44%), as a potential role in biocontrol and metal acquisition in the cacao rhizosphere.

## Data Availability

The genome is at DDBJ/ENA/GenBank under the genome assembly ASM4394422v1. The accession number is JBIMPM010000000. The SRA is SRR32762106. The BioProject ID is PRJNA1173161, and the BioSample ID is SAMN44293728.
